# Moniletrix of the scalp from almost normal aspect to total alopecia: variable intrafamilial expressiveness^☆^^☆☆^

**DOI:** 10.1016/j.abd.2020.07.019

**Published:** 2021-07-14

**Authors:** Daniela Antoniali, Andrezza Telles Westin, Fernanda André Martins Cruz, João Carlos Lopes Simão

**Affiliations:** Division of Dermatology, Department of Internal Medicine, Faculty of Medicine, Universidade de São Paulo, Ribeirão Preto, SP, Brazil

**Keywords:** Alopecia, Hair diseases, Hypotrichosis, Monilethrix

## Abstract

Monilethrix is a rare defect of the hair shaft, with most cases showing an autosomal dominant pattern of inheritance and variable clinical expression. It is characterized by hypotrichosis secondary to hair fragility. The diagnosis is made through trichoscopy, detecting typical findings such as periodic narrowing at regular intervals, giving the hair the appearance of beads in a rosary. This article reports the case of six members of a family diagnosed with monilethrix with alopecia of varying degrees.

## Introduction

Monilethrix is a rare inherited defect, with most cases showing an autosomal dominant pattern, with incomplete penetrance and variable expressiveness. It is characterized by hair shaft dysplasia, resulting in hypotrichosis due to fragility and breakage. Trichoscopy shows pathognomonic findings in the hair shaft, with nodules and constrictions, known as hair shaft with the appearance of ‘rosary beads’. This is the case of a family consisting of six members from two generations who were diagnosed with monilethrix presenting a variable clinical spectra.

## Case report

**Figure 1:**
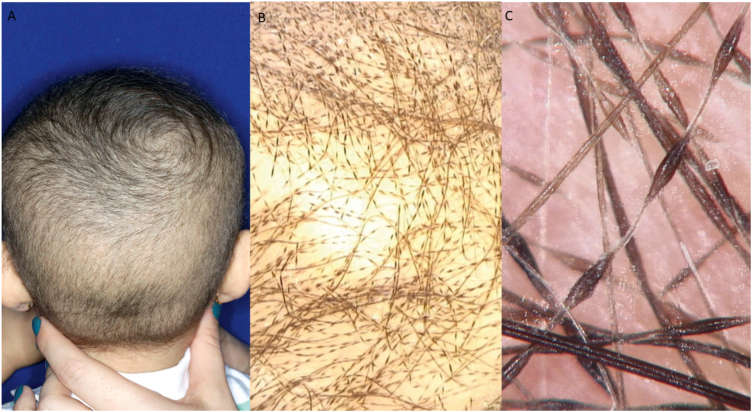
One-year-old girl. (A), Clinical aspect. (B), Trichoscopy (macroscopic aspect). (C), Trichoscopy (×70 magnification).

**Figure 2:**
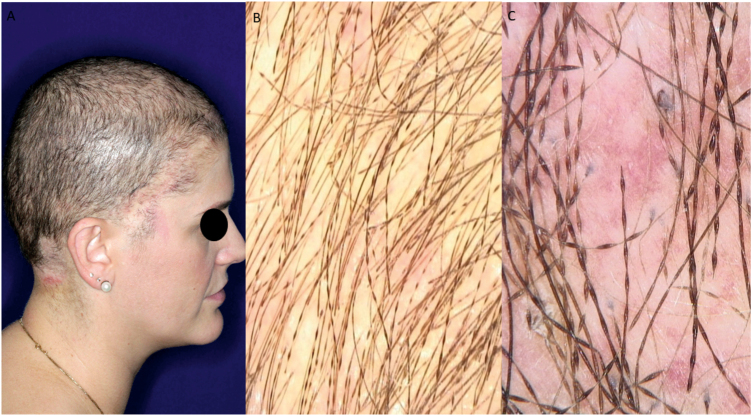
Thirty-four-year-old woman. (A), Clinical aspect. (B), Trichoscopy (macroscopic aspect). (C), Trichoscopy (×70 magnification).

**Figure 3:**
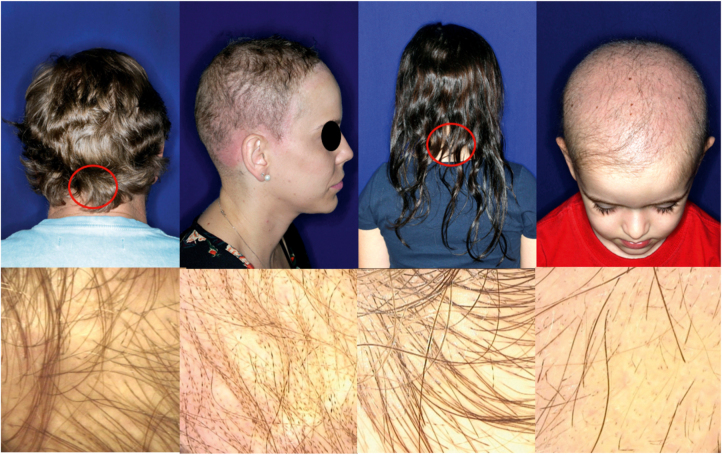
Clinical aspects and their respective trichoscopy. Red circles: affected regions in patients with lesser involvement.

**Figure 4:**
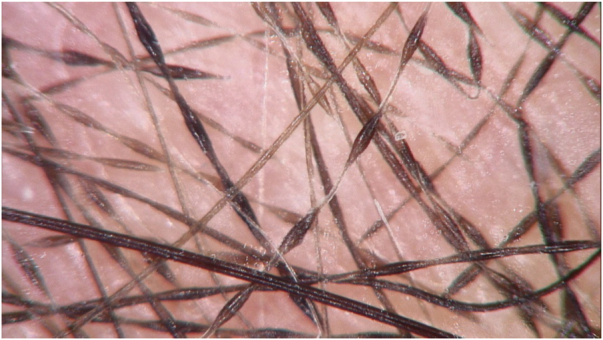
Trichoscopy with regular variations in the diameter of the hair shaft, with elliptical dilations and constrictions (×70 magnification).

**Figure 5:**
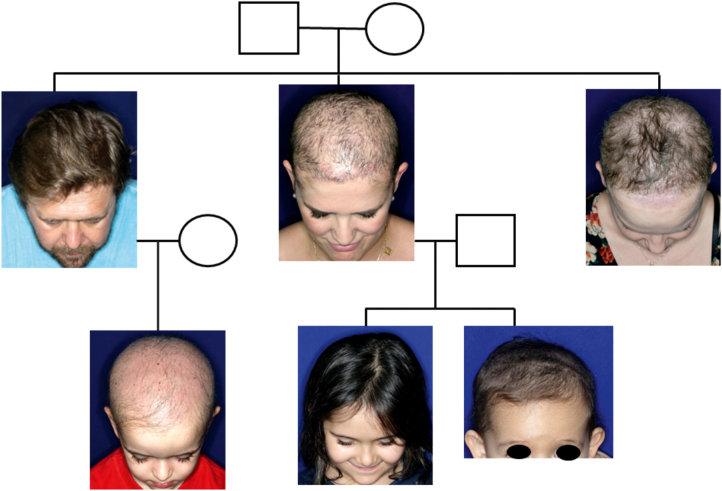
Heredogram of the reported family.

**Figure 6:**
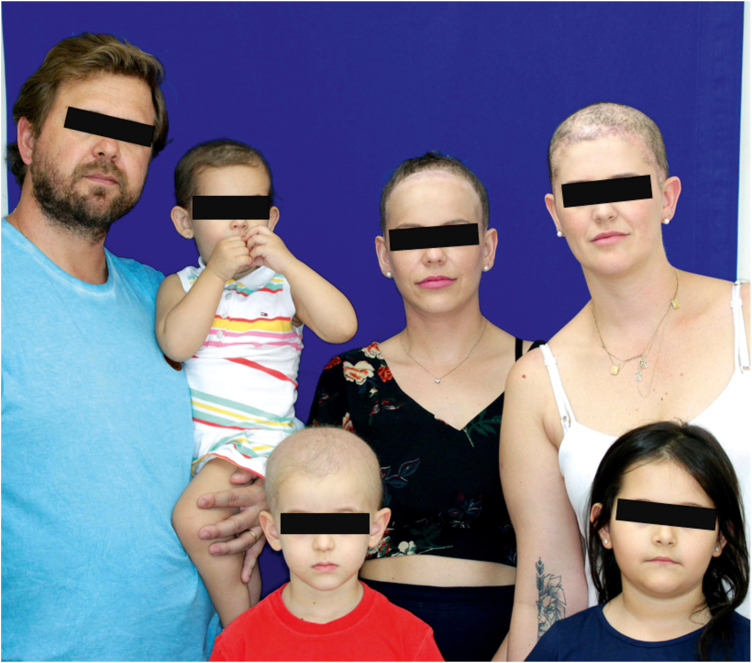
Variability of the clinical expression of monilethrix in the affected family members.

A one-year-old girl with hair thinning throughout the scalp since birth, accompanied by a five-year-old sister with a history of diminished hair growth in the occipital region (Fig. 1). The mother reported a diagnosis of monilethrix, with different degrees of clinical expressiveness and severity in the family. The affected members were the children's own mother (34-year-old - Fig. 2), the maternal uncles (a 37-year-old man and a 30-year-old woman - Fig. 3), and a male cousin (four-year-old - Fig. 3). The children's physical examination and neuropsychomotor development were normal. Dermatological examination of the youngest child revealed short thin hair shafts, more evident in the occipital region. Trichoscopy of the scalp revealed hairs with regularly spaced elliptical nodules, separated by constrictions, some of them fractured (Fig. 4). The dermatological examination of the five-year-old child revealed long hair with only a circumscribed occipital area of diminished hair density. Trichoscopy of the entire scalp was normal, except for the occipital area, which showed hairs with regularly spaced elliptical nodules, separated by intermittent internodes. Neither of the children had any alterations on the nails, eyebrows and eyelashes, and the traction test was negative. The clinical and trichoscopy findings allowed the diagnosis of monilethrix. All affected family members came to the dermatological unit for an in-person assessment of the disease spectrum involvement (Figure 5, Figure 6).

## Discussion

Monilethrix is a rare hereditary condition characterized by abnormalities in the hair shaft, which may have an autosomal dominant (mutations in the KRT81, KRT83 and KRT86 genes associated with trichokeratins) or autosomal recessive pattern of inheritance (mutation in the desmoglein 4 gene with malformation of the hair shaft desmosomes)^1^, ^2^ The term moniletrix comes from *monile* (necklace, in Latin) and *thrix* (hair, in Greek). In this condition, the hair shaft is characterized by periodic narrowing at regular intervals, giving the hair the appearance of beads in a rosary.^3^ The wide or nodular part of the hair corresponds to the normal thickness of the shaft containing the medulla and the internodal narrowing corresponds to the anomaly, with the absence of the medulla being the cause of the shaft thinning. The nodules are elliptical, separated by 0.7 to 1.0 mm intervals showing normal thickness or a slightly lower than the normal shaft diameter. Fragility and fractures can be observed at the constriction points.^4^

There is considerable variation in the clinical expressiveness and there is usually intrafamilial variability. The disease can range from an almost normal scalp, with few alterations, to total alopecia.^5^ The milder forms can go unnoticed, with few affected hairs. In severe cases, secondary sexual hairs, as well as eyebrows and eyelashes may be compromised.^3^ Hair alterations can occur alone or are associated with keratosis pilaris, syndactyly, cataracts, dental and nail abnormalities, and can determine cicatricial alopecia.^3^, ^6^

Families that were investigated for monilethrix show there is no correlation between the disease genotype and the phenotype. This means the same mutation can be expressed with different degrees of severity and it raises the question of whether there are other factors influencing this mutation, including environmental and other non-genetic factors.^7^ At birth, the hair looks normal; however, it is progressively replaced by hair shafts with alterations, becoming fragile and brittle within the first months of life.^3^ Diffuse hypotrichosis occurs, which sometimes is very severe. There is a greater number of abnormal hair shafts in the temporal and occipital regions, in comparison with the rest of the scalp, as they are areas that are more exposed to friction. ^1^ In the most severe cases, hairs can be extremely short due to fractures that occur soon after follicle eminence or even inside the follicular ostium. Fractured shafts can rupture the outer root sheath and cause foreign body granulomas, clinically corresponding to erythematous papules or follicular hyperkeratosis.^1^ Trichoscopy shows regular variations in the hair shaft diameter, with elliptical dilations (nodes) and constrictions (internodes).^6^

There is no definitive treatment for moniletrix. Case reports have shown improvement with topical use of 2% Minoxidil and systemic biotin, N-acetyl cysteine, corticosteroids, acitretin, oral contraceptives, and griseofulvin. 8, 9 Spontaneous improvement can occur with age. The most important is to inform parents and patients about heredity, the evolution of the condition, prognosis, and how to prevent trauma to the hair shafts. Patients should be advised to gently handle hair as little as necessary, abuse of conditioning agents during washing and after it, using leave-in products, in addition to being careful when using hair ornaments and adornments that might cause traction.^10^

## Financial support

None declared.

## Authors’ contributions

Daniela Antoniali: Conception and planning of the studied case; intellectual participation in the propaedeutic and/or therapeutic conduct of the studied case; review of the literature; drafting and editing of the manuscript.

Andrezza Telles Westin: Drafting and editing of the manuscript; collection, analysis, and interpretation of data; intellectual participation in the propaedeutic and/or therapeutic conduct of the studied case; critical review of the literature; critical review of the manuscript.

Fernanda André Martins Cruz: Approval of the final version of the manuscript; drafting and editing of the manuscript; participation in research orientation; intellectual participation in the propaedeutic and/or therapeutic conduct of the studied case; critical review of the literature; critical review of the manuscript.

João Carlos Lopes Simão: Approval of the final version of the manuscript; drafting and editing of the manuscript; participation in research orientation; intellectual participation in the propaedeutic and / or therapeutic conduct of the studied case; critical review of the literature; critical review of the manuscript.

## Conflicts of interest

None declared.
